# New Treatments for Spinal Nerve Root Avulsion Injury

**DOI:** 10.3389/fneur.2016.00135

**Published:** 2016-08-23

**Authors:** Thomas Carlstedt

**Affiliations:** ^1^Wolfson CARD, Kings College London, London, UK

**Keywords:** nerve plexus, root avulsion, neurotization, adjuvant therapy, sensory recovery, spinal cord regeneration

## Abstract

Further progress in the treatment of the longitudinal spinal cord injury has been made. In an inverted translational study, it has been demonstrated that return of sensory function can be achieved by bypassing the avulsed dorsal root ganglion neurons. Dendritic growth from spinal cord sensory neurons could replace dorsal root ganglion axons and re-establish a reflex arch. Another research avenue has led to the development of adjuvant therapy for regeneration following dorsal root to spinal cord implantation in root avulsion injury. A small, lipophilic molecule that can be given orally acts on the retinoic acid receptor system as an agonist. Upregulation of dorsal root ganglion regenerative ability and organization of glia reaction to injury were demonstrated in treated animals. The dual effect of this substance may open new avenues for the treatment of root avulsion and spinal cord injuries.

## Introduction

In a previous communication, the basic science background and its clinical translation to the first surgical method that results in functional return after a spinal cord injury was given (1). The traumatic tear of nerve roots from the spinal cord interrupts the segmental transverse sensory and motor nerve fibers causing a longitudinal spinal cord injury. If left untreated, the affected spinal cord segments can deteriorate over about 1 month, with disintegration of neuronal networks and death of motor (2), sensory (3), and autonomic (4) nerve cells. The clinical effect of such lesion is loss of motor and sensory and autonomic function. Such injury in the human occurs most frequently in road traffic accidents affecting mainly the nerve plexus formations for limb function mostly for the arm, i.e., the origin of the brachial plexus. The cure of this condition depends on regeneration in the central nervous system (CNS).

By reimplanting avulsed nerve roots into the spinal cord functional useful motor but not sensory function is restored if the procedure is performed before 1 month after injury. This unique surgical strategy has since 25 years been a routine procedure in centers for brachial plexus injury in Stockholm and London (5). Close to 100 patients with at least subtotal brachial plexus root avulsions have been treated. In most patients, there is motor recovery which is functional in about 70% of the cases (6). Like in other types of brachial plexus or proximal nerve injuries mainly the proximally situated muscles recover good function. Distal muscles such as in the hand rarely regain any useful activity, although this has been described in some patients (7, 8). Remaining problems with this technique are injury-induced neuronal death, direction and specificity of reinnervation, and muscle and sensory receptor disintegration from long time of denervation.

Sensory recovery is not possible to restore only by reimplanting avulsed dorsal roots. The dorsal root ganglion neurons are unable to regenerate into the adult spinal cord (9). The molecular and cellular events that repel or arrest axons in the PNS–CNS transitional region remain elusive, but the idea that axons may terminate regeneration by synapsing with non-neuronal cells was originally proposed (9). This provocative idea has recently been supported (10). Proteoglycan producing NG2 cells have been indicated to participate in such process (11), and it is therefore pertinent to further study these cells in conjunction with dorsal root de- and regeneration across the PNS–CNS transitional region (see below). Since the previous communication (1), experimental and clinical studies have been performed in order to overcome the problem of sensory recovery after dorsal root avulsion injury.

## Intramedullary Neuron Transfer to Re-Establish Sensory Function

A type of palliative “neurotization” procedure to replace the avulsed dorsal root and its ganglion containing the primary sensory neurons was developed. The concept of ventral root or nerve graft implantation to promote growth from spinal cord motoneurons to the periphery was the basis for a procedure to reconnect the periphery with spinal cord sensory systems. Sensory nerve cells in the spinal cord could hypothetically in accordance with CNS motoneurons potentially elongate new processes into a PNS graft implanted into the dorsal spinal cord to reconnect with the periphery. When asking spinal cord neurons to extend new processes into the PNS, it is likely that not axons but dendrites would be recruited by the PNS conduit implanted into the dorsal horn. It is well established that dendrites can produce aberrant or supernumerary axons after injury. Such processes have been shown to extend from dendrites into the PNS and are called dendraxons (12) or unusual distal processes (UDP) (13). Extension of supernumerary axons or dendraxons into a PNS conduit has previously been demonstrated for motoneurons (12, 14). It has also been shown that such processes can transmit impulses and contain transmitter substances for synaptic communication (14).

Experimentally, it was shown that intrinsic sensory spinal cord neurons can extend new (non-regenerative) processes into an implanted PNS conduit. Immunohistochemical technique (MAP2 staining) showed that these neurites were dendrites that had extended into the implanted PNS conduit and have functional properties (9). Electrophysiology verified that the new growth from sensory spinal cord neurons can transmit impulses. There was also a demonstration of transsynaptically provoked muscle contraction when stimulating these neurites which demonstrates that an integration of this new growth of spinal cord neurons with segmental spinal cord circuits in particularly ventral horn motoneurons occurred.

The implantation of a PNS conduit into both the ventral for motor recovery and dorsal part of the spinal cord for sensory recovery was performed in clinical cases of brachial plexus avulsion injury. Following such procedures, proprioception together with muscle function could be demonstrated (8). The biceps tendon reflex was confirmed clinically and H-reflex by means of electrophysiology (8). This is intriguing as proprioceptive function has been demonstrated to disappear and not to recover after a nerve injury with regeneration (15). With this extended spinal cord surgery including also sensory neurons, it was obvious that movements had become more controlled without the usual synkinesis seen in cases were motor conduits only had been reconstructed. In contrast to recovery of proprioception, there were no clinical, electrophysiological, or structural signs of exteroception (8).

## Adjuvant Therapy for Spinal Cord Sensory Regeneration

The dorsal root injury is considered as a type of spinal cord injury and as such the most common spinal cord injury (16). The palliative neurotization procedure described earlier has not resulted in a full sensory recovery. It is obvious that adjuvant therapy is necessary to complement surgery in order to recover better sensation after dorsal root injury or avulsion from the spinal cord. There are at least two major reasons for the failure of injured dorsal root axons to regrow back into the spinal cord. There is a lack of intrinsic neuronal growth, based largely on inactivity in the phosphoinositide3-kinase (PI3K)/Akt/mammalian target of rapamycin (mTOR) pathway, which is negatively regulated by phosphatase and tensin homolog (PTEN) (17). Another major impediment to regeneration into the spinal cord is the formation of an inhibitory environment by a glia scar.

The retinoic acid signaling system is very powerful in neuron growth and regeneration. Previous work has shown that retinoic acid receptor (RAR) beta 2 signaling stimulates axonal outgrowth in human, mouse, and rat (18, 19). In the adult, retinoic acid-responsive elements are locally activated in the regenerating rat nerve after a peripheral nerve injury (20). In adult spinal cord explants and dorsal root ganglion neurons where RARbeta 2 is absent, overexpression of RARbeta stimulates neurite outgrowth (21). Furthermore, delivery of RARbeta 2 to adult neurons induces axonal regeneration programs within injured neurons and encourages axonal growth also in the inhibitory CNS (22).

A small lipophilic molecule which is an agonist to the nuclear receptor RAR beta has been developed to be used in conjunction with dorsal root to spinal cord reimplantation surgery. In a rat model of cervical dorsal root avulsion (C5–T1), the newly developed RARbeta agonist was given (23). After 4 weeks of treatment, behavioral tests showed recovery of sensory (the sticky tape test; sensing and removal) and locomotor function (the horizontal ladder test). Dorsal root fiber regeneration across the dorsal root peripheral–central transitional region (PNS–CNS TR) was shown by biotinylated dextran amine (BDA) labeling and by means of tractography which showed a robust ingrowth of neurites. Synaptic recovery was demonstrated by analysis of noxious heat stimuli response, synaptic density, and mechano- and proprioceptive synapses in the dorsal horn showing that new connections had been established in the spinal cord (23).

Of paramount importance is how the regenerated dorsal root axons re-entered the spinal cord. In the naive adult situation, there is a specialized transitional region between a stereotype peripheral nerve compartment of the dorsal root and the central nervous fiber tracts of the spinal cord, which involves a number of unique structural entities (24). Among these are the occurrences of fibrous astrocytic processes surrounding and separating the most proximal peripheral paranodes and a compound PNS–CNS type of node of Ranvier at the crossing of nerve fibers from the PNS to the CNS of organization (25). In the treated animals, a glia construct of a similar organization had been re-established at the passing of regenerated dorsal root axons into the spinal cord. This is of conceptual importance as it seems as a naive structural glia organization has to be repeated to allow a successful regeneration. This is presently the subject of further studies in particular what the role of the NG2 cells are in this context.

Studies of the mechanisms of the switch from a non-permissive environment in the CNS and an increased regenerative activity in the dorsal root neurons to allow for regeneration demonstrated that the RARbeta agonist modulates the PTEN signaling pathway in both neurons and astrocytes. In neurons RARbeta, *via* a cytoplasmic effect, induces PTEN to move from the membrane where it blocks axonal growth *via* the PI3K inhibition (17), into the cytoplasm, where it becomes phosphorylated and hence inactive (26). In addition, stimulation of RARbeta results in an increased secretion of PTEN in exosomes. These are taken up by astrocytes, resulting in hampered proliferation and glia scar formation as well as causing them to arrange in a normal-appearing scaffold around the regenerating axons allowing them to grow back into the spinal cord (23). The dual effect of RARbeta signaling, both neuronal and neuronal–glial, results in axonal regeneration in the spinal cord after dorsal root injury.

In summary, there is the potential of new growth and plasticity rather than regeneration of spinal cord sensory neurons that can replace the injured primary sensory dorsal root neurons and reconnect to the periphery for reestablishment of some but not all sensory qualities. In order for a more complete return of sensory function after dorsal root avulsion from the spinal cord, a unique adjuvant therapy has been developed.

## Author Contributions

The author confirms being the sole contributor of this work and approved it for publication.

## Conflict of Interest Statement

The author declares that the research was conducted in the absence of any commercial or financial relationships that could be construed as a potential conflict of interest.
