# Corrigendum: New Treatments for Spinal Nerve Root Avulsion Injury

**DOI:** 10.3389/fneur.2017.00326

**Published:** 2017-07-05

**Authors:** Thomas Carlstedt

**Affiliations:** ^1^Wolfson CARD, Kings College London, London, United Kingdom

**Keywords:** nerve plexus, root avulsion, neurotization, adjuvant therapy, sensory recovery, spinal cord regeneration

Missing Funding:

In the original article, I neglected to include the funders: Wellcome Trust, Karolinska Institutet, Swedish Defence FOT-AF.9221006, and Darwin Trust of Edinburgh. The author apologizes for this error and state that this does not change the scientific conclusions of the article in any way.

Text Correction:

In the original article, there were two errors with the inclusion of the texts:

“Pharmacokinetic and pharmacodynamics studies have demonstrated dose dependent efficacy in oral administration of the RARbeta agonist. Toxicological tests have shown little adverse effects. Clinical trials are now imminent in the most experienced centers for plexus injuries (London and Stockholm) using this first drug that can be given orally for direct treatment of a spinal cord injury.”

and

“A new drug that can be given orally restores sensory functions and muscle coordination after such injury by means of regeneration within the spinal cord. If successful in clinical trials this drug can be considered for many other CNS injuries.”

Corrections by deleting those texts have been done to section “Adjuvant therapy for spinal cord sensory regeneration” paragraph six and seven.

The author apologizes for this error and state that this does not change the scientific conclusions of the article in any way.

The original article has been updated.

## Conflict of Interest Statement

The author declares that the research was conducted in the absence of any commercial or financial relationships that could be construed as a potential conflict of interest.

